# Supplementary material for: MetaRibo-Seq measures translation in microbiomes
Source: Nat Commun. 2020 Jun 29;11:3268. doi: 10.1038/s41467-020-17081-z (PMC7324362; doi:10.1038/s41467-020-17081-z)
Supplement: Supplementary file 10 — Supplementary Data 7 [file 41467_2020_17081_MOESM10_ESM.zip › File2/Confidence_High_Taxonomy/179793_out.krona.html]

Javascript must be enabled to view this page.

members
magnitude
magnitudeUnassigned
count
unassigned
taxon
rank

179793\_out

11

2
superkingdom
11

1239
11
phylum

class
11
186801

186802
11
order

1
family
31979

genus
1
1485

1

SRS051031\_contig\_number\_23320
species
1262815

10

SRS013687\_contig\_number\_25790SRS016095\_contig\_number\_20872SRS016541\_contig\_number\_10974SRS023914\_contig\_number\_22115SRS050801\_contig\_number\_126SRS051031\_contig\_number\_35588SRS053356\_contig\_number\_28567SRS077392\_contig\_number\_25640SRS098571\_contig\_number\_42818SRS1041145\_contig\_number\_27882
species
1898207
